# Co-methylation networks associated with cognition and structural brain development during adolescence

**DOI:** 10.3389/fgene.2024.1451150

**Published:** 2025-01-07

**Authors:** Dawn Jensen, Jiayu Chen, Jessica A. Turner, Julia M. Stephen, Yu-Ping Wang, Tony W. Wilson, Vince D. Calhoun, Jingyu Liu

**Affiliations:** ^1^ Tri-Institutional Center for Translational Research in Neuroimaging and Data Science (TReNDS): (Georgia State University, Georgia Institute of Technology, and Emory University), Atlanta, GA, United States; ^2^ Neuroscience Institute, Georgia State University, Atlanta, GA, United States; ^3^ Department of Computer Science, Georgia State University, Atlanta, GA, United States; ^4^ Department of Psychiatry and Behavioral Health, Wexnar Medical Center, Ohio State University, Columbus, OH, United States; ^5^ The Mind Research Network, Albuquerque, NM, United States; ^6^ Department of Biomedical Engineering, Tulane University, New Orleans, LA, United States; ^7^ Institute for Human Neuroscience, Boys Town National Research Hospital, Omaha, NE, United States; ^8^ Psychology Department and Neuroscience Institute, Georgia State University, Atlanta, GA, United States

**Keywords:** adolescent development, methylation, neuroimaging epigenetics, co-methylation, cognition, brain development

## Abstract

**Introduction:**

Typical adolescent neurodevelopment is marked by decreases in grey matter (GM) volume, increases in myelination, measured by fractional anisotropy (FA), and improvement in cognitive performance.

**Methods:**

To understand how epigenetic changes, methylation (DNAm) in particular, may be involved during this phase of development, we studied cognitive assessments, DNAm from saliva, and neuroimaging data from a longitudinal cohort of normally developing adolescents, aged nine to fourteen. We extracted networks of methylation with patterns of correlated change using a weighted gene correlation network analysis (WCGNA). Modules from these analyses, consisting of co-methylation networks, were then used in multivariate analyses with GM, FA, and cognitive measures to assess the nature of their relationships with cognitive improvement and brain development in adolescence.

**Results:**

This longitudinal exploration of co-methylated networks revealed an increase in correlated epigenetic changes as subjects progressed into adolescence. Co-methylation networks enriched for pathways involved in neuronal systems, potassium channels, neurexins and neuroligins were both conserved across time as well as associated with maturation patterns in GM, FA, and cognition.

**Discussion:**

Our research shows that correlated changes in the DNAm of genes in neuronal processes involved in adolescent brain development that were both conserved across time and related to typical cognitive and brain maturation, revealing possible epigenetic mechanisms driving this stage of development.

## 1 Introduction

Considered the second-most critical phase of neurodevelopment in humans, adolescence is marked by improved cognitive performance driven by widespread reorganization of the brain (Steinberg, 2005). Animal studies have shown that there are large-scale epigenomic changes happening during this phase of heightened synaptogenesis (Mychasiuk and Metz, 2016), but we are still just beginning to understand what role epigenetics plays in the development of the adolescent human brain as well as the associated cognitive improvements (Wheater et al., 2020). Until recently, the bulk of this research in humans had been restricted to fetal brain development, limited by the need to directly analyze brain tissue (Schneider et al., 2016). Advancements in analysis tools have made it possible to study the epigenetic mechanisms, such as DNA methylation (DNAm), of neural development using peripheral tissue samples such as blood or saliva (Walton et al., 2016; Lin et al., 2018; Proskovec et al., 2020). Methylation of DNA occurs when a methyl group attaches to a cytosine pyrimidine (CpG) ring (Mangiavacchi et al., 2023; Moore et al., 2013; Perri et al., 2017). This acts as one mechanism of gene expression regulation, for example, when DNAm at a promoter region reduces overall transcription of a downstream gene, decreasing its expression or causing alternative splicing (Dupont et al., 2009).

Biomarkers of DNAm in peripheral tissue such as saliva and blood have been directly associated with DNAm in brain tissues (Braun et al., 2019a; Han et al., 2019). These peripheral tissue measures have been associated with both structural and functional aspects of the brain (Walton et al., 2016; Lin et al., 2018; Proskovec et al., 2020; Braun et al., 2019a). Researchers using resected brain tissue from 27 subjects, as well as their saliva, blood, and buccal samples, established that individual CpG sites had high correspondence across tissue-types as well as an epigenome-wide correlation between tissues as high as 0.90 (Braun et al., 2019a). With this advancement, research into the epigenomic mechanisms of adolescent development have expanded. A 2019 study found significant change in the DNAm of 15k CpGs pre- and post-adolescence from blood samples taken from a population spanning 10–18 years of age (Han et al., 2019). A study published in 2021 found that measures of DNAm from blood samples significantly mediated the relationship between childhood adversity and symptoms of depression across adolescence (Smith et al., 2021). Investigation of correspondence of DNAm in surrogate tissues (blood and saliva) as biomarkers for DNAm in other places in the body extends beyond the brain. Research published in 2020 has found that DNAm in blood reliably corresponds to DNAm in bone tissues (Ebrahimi et al., 2020), which like the brain, requires invasive procedures to ascertain directly. In 2024, a group of researchers has also found strong associations between DNAm in blood and DNAm regulation of genes involved in the brain associated with Alzheimer’s disease (Mendonça et al., 2024). They also found differential DNAm change in the blood of patients with Parkinson’s that were strongly related to DNAm changes on genes mechanistically related to Parkinson’s, demonstrating that DNAm changes in peripheral tissue can be related to different disease states, suggesting that this correspondence between tissues is not coincidental (Mendonça et al., 2024).

Our own previous research, using DNAm measures from saliva, found that DNAm changes at seven CpGs located on genes involved with excitatory and inhibitory mechanisms (*GRIN2D, GABRB3, KCNC1, SLC12A9, CHD5, STXBP5,* and *NFASC)* were significantly associated with grey and white matter maturation, as well as with cognitive development during adolescence (Jensen et al., 2023a; Jensen et al., 2023b). Those only included a small selection of CpGs, so to further expand our understanding of epigenetic influences on normal cognitive and brain development during adolescence, our current study uses a weighted gene correlation network analysis (WGCNA) (Langfelder and Horvath, 2008) to explore the epigenome-wide mechanisms. The modules created using this WGCNA highlight interconnected genomic regions based on correlated methylation levels, which are clustered into biologically relevant networks (Langfelder and Horvath, 2008).

Neural and cognitive development in adolescence is fairly well documented. Multiple longitudinal MRI studies have shown that grey matter volume (GM), as measured by structural MRI (sMRI) decreases non-linearly (Gogtay et al., 2004) from the onset of adolescence, followed by a slightly delayed increased in myelination, which is reflected as an increase in fractional anisotropy (FA), measured using diffusion MRI (dMRI) (Bava et al., 2010). This structural and functional reorganization of the brain is accompanied by improvement across a broad spectrum of cognitive measures that include attention, memory and processing speed (Shaw et al., 2006).

To explore how these correlated networks of DNAm might be related to adolescent maturation, we used data collected from the Developmental Chronnecto-Genomics (Dev-CoG): A Next-Generation Framework for Quantifying Brain Dynamics and Related Genetic Factors in Childhood, which is a longitudinal cohort of roughly 200 typically developing subjects aged 9–14 (Stephen et al., 2021). This project collected brain imaging, cognitive assessments, and saliva for DNAm analysis over three time points, with roughly 1 year between each collection (Stephen et al., 2021). Using this data in a previous study, we identified a small subset of CpGs strongly related to cognitive development, grey and white matter maturation (Jensen et al., 2023a; Jensen et al., 2023b).

The purpose of this study is to further expand on this initial exploration by quantifying the relationships between co-methylation networks, identified using a weighted correlation network analysis, and neural and cognitive development in adolescence. These co-methylation networks, representing clusters of CpGs interconnected based on the changes in their methylation across time, will be included in a multivariate analysis of covariance to investigate the relationships between these networks of correlated DNAm change, networks of GM volume and FA changes, and the improvements on cognitive tests. We hypothesize that we will identify modules of correlated DNAm changes at CpGs on genes that will highlight biologically relevant pathways significantly related the maturation of grey matter, white matter, and cognition.

## 2 Materials and methods

### 2.1 Cohort

The same cohort of subjects from our previous work (Jensen et al., 2023a) was used in this analysis, recruited at the Mind Research Network (MRN) and the University of Nebraska Medical Center (UNMC) as part of the Dev-CoG study (Stephen et al., 2021), approved by the relevant institutional review board at each data collection site (Advarrra IRB–MRN and UNMC IRB–Nebraska). Data sharing was written into the consent forms and the study protocols (Stephen et al., 2021). The inclusion criteria for the study were: English speaking, age 9–14 years at enrollment and both child and parent were able and willing to assent/consent to the study. The exclusion criteria for the study were: current pregnancy, unable to consent/assent, history of developmental delays or disorders (or an individual education plan indicative of a developmental delay/disorder), history of epilepsy or other neurological disorders, parental history of major psychiatric or neurological disorder, self-reported prenatal exposure to alcohol or drugs, medication use, contraindication to MRI (MRI screening form was reviewed), or metal orthodontia (e.g., braces or spacers) (Stephen et al., 2021). Images, saliva samples, and cognitive tests were collected from 200 participants over three time points, roughly a year apart. See Table 1 for demographic information. Due to participant dropout during longitudinal data collection, our deltaT1 and deltaT2 analyses have different sample sizes. The multivariate analyses of differences between time points included 145 subjects (mean baseline age 11.71 years old, 75 females, 70 males) for deltaT1, and 81 subjects (37 females, 44 males) for deltaT2. To account for possible bias introduced by the attrition, a chi-square test was performed between deltaT1 and deltaT2 biased the groups with regards to gender—there was no significant difference in the ratio of genders between the deltaT1 and deltaT2. Similar tests were performed for SES and race, with no significant differences found between groups. A *t*-test also established that there were no significant differences in the distribution of the baseline ages between groups.

**TABLE 1:** General demographic information.

Demographics	MRN (101)	UNMC (102)
Mean age at enrollment (range)	11.3 (9–14)	11.2 (9–14)
Gender (M/F)	51M/50F	51M/51F
Race (Caucasian/BIPOC)	86/15	87/15
Ethnicity (% Hispanic)	41.6%	7.8%
Mean WASI-II IQ (Range)	108.6 (72–139)	112.1 (68–148)
Mean SES (Range)	42.6 (17–66)	48.2 (15–65)

MRN, Mind Research Network; UNMC, University of Nebraska Medical College; BIPOC, Black, Indigenous, and People of Color; WASI-II IQ, Wechsler Abbreviated Scale of Intelligence; SES, Socioeconomic Score.

### 2.2 DNA methylation preprocessing

The preprocessing largely followed the ENIGMA epigenetics protocol and was used in our previous studies (Jensen et al., 2023a; Jensen et al., 2023b). DNAm from saliva was assessed for each subject using the Illumina HumanMethylation850 (850k) microarray, which measures CpG methylation across ∼850,000 probes covering 99% of gene promoters. Standardized quality control procedures and quantile normalization was performed using the minfi Bioconductor package in R (version 3.6.2) (Aryee et al., 2014). Red and green channel intensities were mapped to the methylated and unmethylated status, samples were checked against the mean intensity to identify low quality. Beta values, calculated for each CpG, for each subject, reflect the degree of methylation using a range of zero, meaning no methylation, to one, meaning completely methylated. To identify outliers, a principal component analysis (PCA) was performed on the beta values. Any sample with values more than three standard deviations away from the median on any of the first four components was removed, as were samples where the genetically determined sex differed from self-report. 20 duplicate DNA samples were included in each batch and checked to ensure measurement reliability. Samples processed in different batches were merged at this stage. Stratified quantile normalization was then applied across samples, using the minfi PreprocessQuantile function. The cell proportions for each DNAm sample were calculated by implementing the estimateCellCounts function in minfi, using our modified reference panel of five types of blood cells (B cells, CD8T and CD4T cells, NK-LGL cells, monocytes, and granulocytes) and epithelial cells (GSE46573) (Zheng et al., 2018). The proportion of total blood cells and epithelial cells was strongly in alignment with EpiDISH (Zheng et al., 2018) estimated immune cells and epithelial cells (correlation >0.98). The cell type effect was regressed out from all the samples to account for the change of cell proportion over time. Batch effects were then corrected using the R package Combat, which assumes normalized data and equalizes the mean from all batches, making negative values possible (Johnson et al., 2007).

### 2.3 Weighted gene correlation network analysis

After preprocessing, approximately 750K CpG sites were retained. We kept only CpG sites with a standard deviation of 0.1 or higher at the first time point to ensure that methylation variability across subjects exceeded measurement variability (Duan et al., 2021). This resulted in 2,414 CpGs for this analysis. To calculate the rate and amount of change, time point 1 (TP1) was subtracted from time point 2 (TP2) to create the deltaT1 difference map for each individual, and TP2 was subtracted from time point 3 (TP3) to create the deltaT2 difference map. To identify correlation patterns within the methylation data, WGCNA was performed using the R package of the same name (WGCNA v. 3.3.3) (Langfelder and Horvath, 2008). The WGCNA pipeline is as follows: (1) to down-weight weaker correlations between CpGs, a soft threshold is chosen appropriate to the scale-free topology of the data, which is based on the r^2 as well as the mean connectivity (Langfelder and Horvath, 2008). For our analysis, a soft threshold of 10 fit both criteria. (2) Adjacency matrices were computed, representing pairwise correlation coefficients (Pearson’s *r*) transformed by the aforementioned *β* to ensure a scale-free correlation structure. These were unsigned matrices, transforming the absolute values of the coefficients in order to preserve both positive and negative co-methylation relationships. (3) Using the adjacency matrices, topological overlap matrices (TOMs) were computed, representing the interconnectedness between pairs of CpGs, both directly and indirectly, with connection strengths mediated by shared CpG neighbors that are one-step away (Langfelder and Horvath, 2008). The values from the TOMs were used to calculate a dissimilarity distance measure, DistTOM, effectively 1-TOM. (4) Dendrograms were constructed for the 2,414 CpGs based on hierarchical clustering of DistTOM scores using hclust in R. Modules of co-methylated CpGs were then determined using adaptive branch pruning based on minimum cluster size of 12 CpGs and a branch cut height of 0.75. (5) Module eigengenes (ME) were computed, representing the first principal component of methylation at CpGs assigned to a particular module (Langfelder and Horvath, 2007). Linear models were used to check for sex, age, and race effects. The subject loadings for each module were used in our subsequent multivariate analyses. For interpretation of the modules, gene set enrichment was done using Reactome (Gillespie et al., 2022). Reactome is a peer-reviewed, open-source and open access pathway database of metabolic and signaling molecules and their biological processes and pathways, cross referenced to more than 100 online bioinformatic resources that include NCBI Gene, Ensembl, UniProt, UCSC Geneome Browser, and the ChEBI small molecule databases (Gillespie et al., 2022). Genes associated with the CpGs identified in each module (as annotated by the Illumina MethylationEPIC) were entered into the Reactome web-interface. A functional gene network analysis was performed using GeneMANIA (www.genemania.org), a web-based Cytoscape tool developed by Donnelly Centre for Cellular and Biomolecular Research at the University of Toronto (Warde-Farley et al., 2010). To further solidify our interpretation of the results, we conducted a *post hoc* investigation of the cross-tissue correspondence for the CpGs included in the gene enrichment for neuronal pathways using the IMAGE-CpG database (Braun et al., 2019b), which includes saliva-to-brain correspondence.

### 2.4 Structural imaging data

T1-weighted structural MRI (sMRI) images were collected at the MRN site on a Siemens 3T TrioTim scanner, and at UNMC site on a Siemens 3T Magnetom Skyra and Prisma scanners, all with a 32-channel radio frequency coil. Scanning parameters were equilibrated as much as possible. The sMRI images were reoriented and registered to a cohort specific template, created using the ANTS multivariate template generator, and resampled to 2 mm × 2 mm × 2 mm (Andersson et al., 2007a; Andersson et al., 2007b; Sanchez et al., 2012; Avants et al., 2008). Using FAST in FSL, a high-dimensional normalization pipeline, the non-brain tissues were removed, and the grey matter, white matter, and cerebral spinal fluid segmented, leaving normalized, modulated, Jacobian-scaled grey matter images (Zhang et al., 2001) that were smoothed by a 4 mm × 4 mm × 4 mm full width at half maximum Gaussian kernel (Smith and Brady, 1997). The resultant grey matter images then had scanner differences regressed out using a simple linear regression with age and sex as covariates. Two subjects were removed due to movement (framewise displacement from rs-fMRI) above 3 standard deviations from the mean of the group. To calculate the rate and direction of change across time points, grey matter volumes from TP1 were subtracted from TP2 to create the deltaT1 difference map for each individual, and TP2 was subtracted from TP3 to create the deltaT2 difference map. An independent component analysis (ICA) performed via the GIFT toolbox (SBM v1.0b; http://trendscenter.org/software/gift) (Xu et al., 2009) was then applied to the difference maps to extract components/brain networks, where distributed brain regions showed covarying patterns of GM volume changes. The components’ associated loadings reflect these brain regions variation across subjects. Using the minimum description length (MDL) criterion (Calhoun and Adali, 2009), seven components were extracted from the GM volume changes of deltaT1, identifying our brain networks of interest for this study. The direction of the ICA loadings were confirmed through a voxel-based morphometry (VBM) analysis in FSL (Smith et al., 2004), where positive loadings indicate increases in GM volume and negative loadings indicate decreases in GM volume. The spatial maps of these seven components were projected onto the subjects’ deltaT2 GM images to ensure uniformity of comparison. ICA component maps were projected into MNI space for anatomical atlas region identification. Refer to Supplementary Figure 1A to see the complete ICA results for GM, and Supplementary Table 2 for a detailed listing of the brain regions. These regions were identified using the Harvard-Oxford cortical and subcortical structural atlases (Makris et al., 2006; Frazier et al., 2005; Desikan et al., 2006; Goldstein et al., 2007) and the probabilistic cerebellar atlas (Diedrichsen et al., 2009). As shown in our previous study (Jensen et al., 2023a), GM volume decreased across parietal regions and increased in the cerebellum and ventral pre-frontal cortex.

### 2.5 Diffusion imaging data

Diffusion MRI (dMRI) was collected with phase reversed blips. b-null volumes were extracted and used to estimate off resonance fields using FSL (v6.0.3) tool topup (Andersson et al., 2003; Smith et al., 2004). These were used to correct the dMRI volumes for head movement, EPI distortions, and eddy current-induced distortions using FSL tool eddy (Andersson and Sotiropoulos, 2016). Advance motion correction was also performed in eddy to detect motion-induced signal dropout and intra-volume (slice-to-volume) movement (Andersson et al., 2017). Using the AFNI (v.19.1.00) tool *3dDWItoDT*, fractional anisotropy (FA) maps were constructed (Le Bihan et al., 2001). The dMRI derivative images were reoriented and registered to a cohort specific template, created using the ANTS multivariate template generator (Andersson et al., 2007a; Andersson et al., 2007b; Sanchez et al., 2012; Avants et al., 2008). The resultant FA values then had the scanner differences regressed out using a simple linear regression that included age and sex as covariates. To calculate the rate and direction of change over time, the FA values from TP1 were subtracted from TP2 to create the deltaT1 difference map, and TP2 was subtracted from TP3 to create the deltaT2 difference map. An independent component analysis (ICA) built in the GIFT toolbox (SBM v1.0b) (Xu et al., 2009) was then applied to the difference maps to extract components/brain networks, where distributed brain regions showed covarying patterns of longitudinal FA changes. Using the minimum description length (MDL) criterion (Calhoun and Adali, 2009), four components were extracted from the FA changes in deltaT1, identifying our brain networks of interest. The components’ associated loadings reflect the variation of FA change networks across subjects. The direction of the ICA loadings was confirmed using the FSLmeants function to extract the average FA within the component networks, where positive loadings indicated increases in FA and negative loadings indicated decreases in FA. The spatial maps of these four components were projected onto the subjects’ deltaT2 FA images to ensure uniformity of comparison. ICA component maps were projected into MNI space for anatomical atlas region identification. Refer to Supplementary Figure 1B to see the complete ICA results for FA, and Supplementary Table 3 for a detailed listing of the brain regions. These regions were identified using the JHU DTI-based white-matter atlas (Mori et al., 2005; Wakana et al., 2007; Hua et al., 2008) as well as the Harvard-Oxford cortical and subcortical structural atlases (Makris et al., 2006; Frazier et al., 2005; Desikan et al., 2006; Goldstein et al., 2007) and the probabilistic cerebellar atlas (Diedrichsen et al., 2009). Our previous research (Jensen et al., 2023b) showed that FA increased across networks of white matter tracts that include the corpus callosum, parietal, and temporal regions.

### 2.6 Cognitive data

The age-uncorrected standard scores from the following NIH cognitive toolbox tests (Denboer et al., 2014) were collected from each subject: the Picture Sequence Memory (TBPSM) test for 8+ (episodic memory), the Pattern Comparison Processing Speed (PCPS) test for 7+ (processing speed), the Flanker Inhibitory Control and Attention (TBFICA) test for 8+ (executive function), the Dimensional Change Card Sort (TBDCCS) for 8+ (executive function). The Cognition Total Composite Score (COGTC), the Cognition Fluid Composite Score (COGFC) reflecting capacity for new learning, and the Cognition Crystallized Composite Score (COGCC) reflecting past learning were computed. Age-uncorrected scores were used to preserve the sensitivity to differences in age. Scores were corrected for site differences using a linear regression with age and sex as covariates. To calculate the rate of change across time points, scores from TP1 were subtracted from TP2 to create the deltaT1 difference map, and TP2 was subtracted from TP3 to create the deltaT2 difference map. As shown in our previous study (Jensen et al., 2023a), linear mixed-effects repeated measures models confirmed the expected significant improvements in cognitive performance over time (Jensen et al., 2023b). Supplementary Figure 1C highlights the cohort’s improvement in Total cognition across all three time points.

### 2.7 Statistical tests

A multivariate analysis of covariance (MANCOVA) was conducted to explore the relationship between the co-methylation modules and brain maturation. This was performed on data from deltaT1 and deltaT2 separately using the jmv package in R (version 4.1.2) (R: MANCOVA), the subjects’ loadings from seven GM and four FA networks as the dependent variables, the subjects’ loadings for the eigenmodules from the co-methylation analysis as the independent variables, and sex and baseline age as the covariates. MANCOVA results were further tested with linear regression tests for each GM and FA network for potential interactions with sex using the emmeans package in R (version 4.1.2) (RDocumentation, 2024).

Similarly, a multivariate analysis was used to explore the relationship between the cognitive measures and the co-methylation modules. The MANCOVA analysis was performed on data from deltaT1 and deltaT2 separately, where the subjects’ cognitive scores were the dependent variables and the subjects’ loadings for the eigenmodules were the independent variables, with sex and baseline age as covariates.

## 3 Results

### 3.1 Diversity of co-methylation patterns increases over time

**FIGURE 1:**
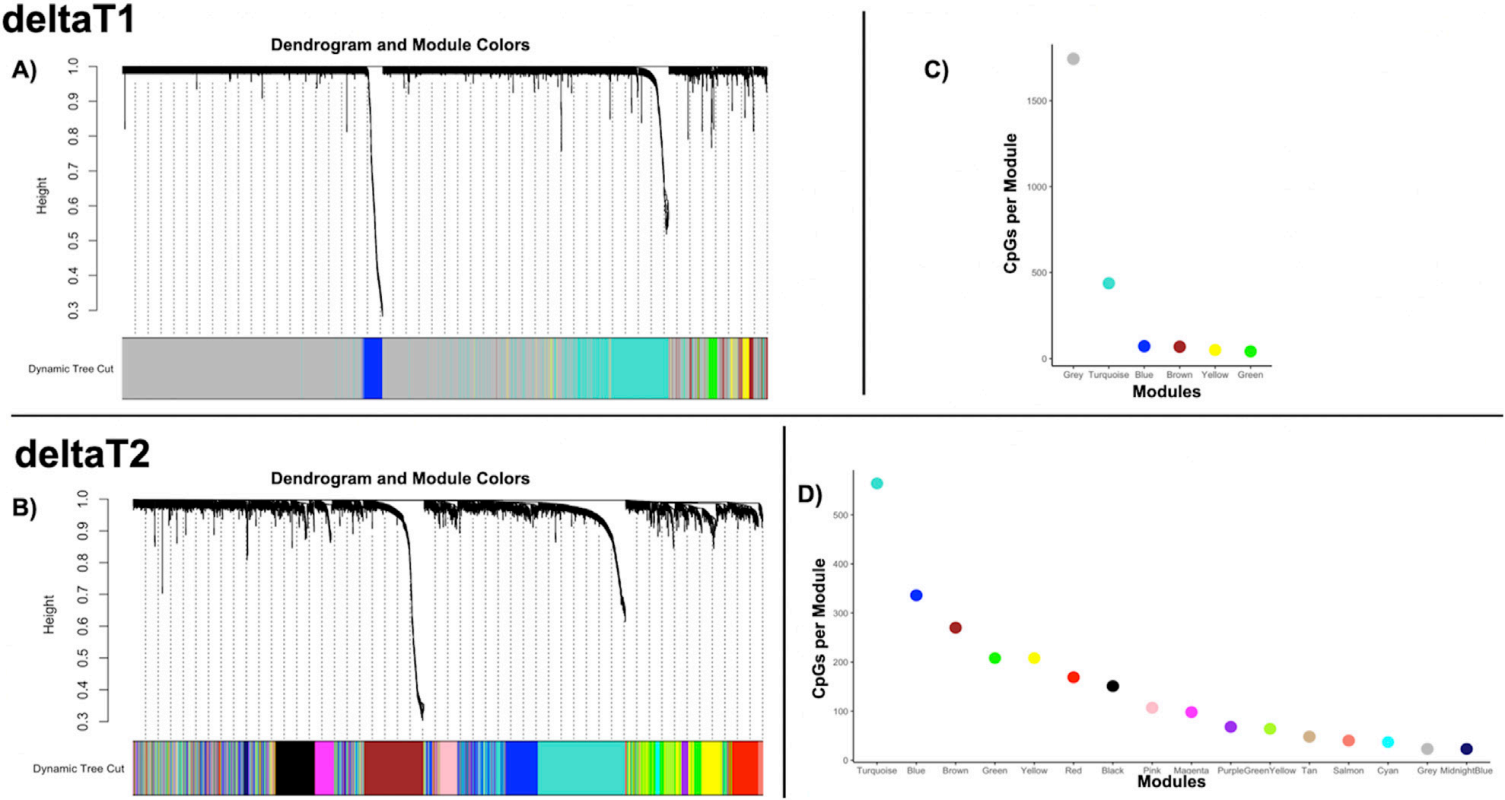
Weighted co-methylation network analysis—**(A, B)** Dendrograms of the WGCNA module assignment for deltaT1 (six modules) and deltaT2 (16 modules) respectively. Each leaf (short vertical lines) in the dendrogram corresponds to a CpG. The branches are modules of highly correlated groups of CpGs with a color (below the dendrogram) to indicate its module assignment. **(C, D)** Graphs of the modules by color, indicating the number of CpGs per module for the WGCNA analysis for deltaT1 and deltaT2 respectively.

From the 2,414 CpGs used in the co-expression analysis, there were six modules of co-methylation identified in deltaT1 and 16 modules of co-methylation in deltaT2 (each deltaT has a grey module, which is the module for non-correlated genes). Figures 1A, B highlight the cluster dendrogram from each deltaT, while Figures 1C, D display the respective module sizes. There were no effects for sex, age, or race in any of the modules from either deltaT. The gene enrichment analysis showed significant results for one module in deltaT1, Blue, where correlated patterns of co-methylation were found enriched in gene transcription, neuronal systems, sodium/proton exchangers, NOTCH signaling, and circadian clock pathways. There were eight modules from deltaT2 with significant gene enrichment results: Blue, Turquoise, Cyan, Brown, MidnightBlue, Tan, Red and Pink. Some pathways with correlated patterns of co-methylation included neuronal systems, gene transcription, immune systems, signal transduction, axon guidance, and NOTCH signaling. For a more extensive list of the gene enrichment analysis for each module, see Supplementary Table 1. No significant results were found for race or social-economic status in any of the modules.

### 3.2 Co-methylation module enriched for neuronal systems conserved across time

**FIGURE 2:**
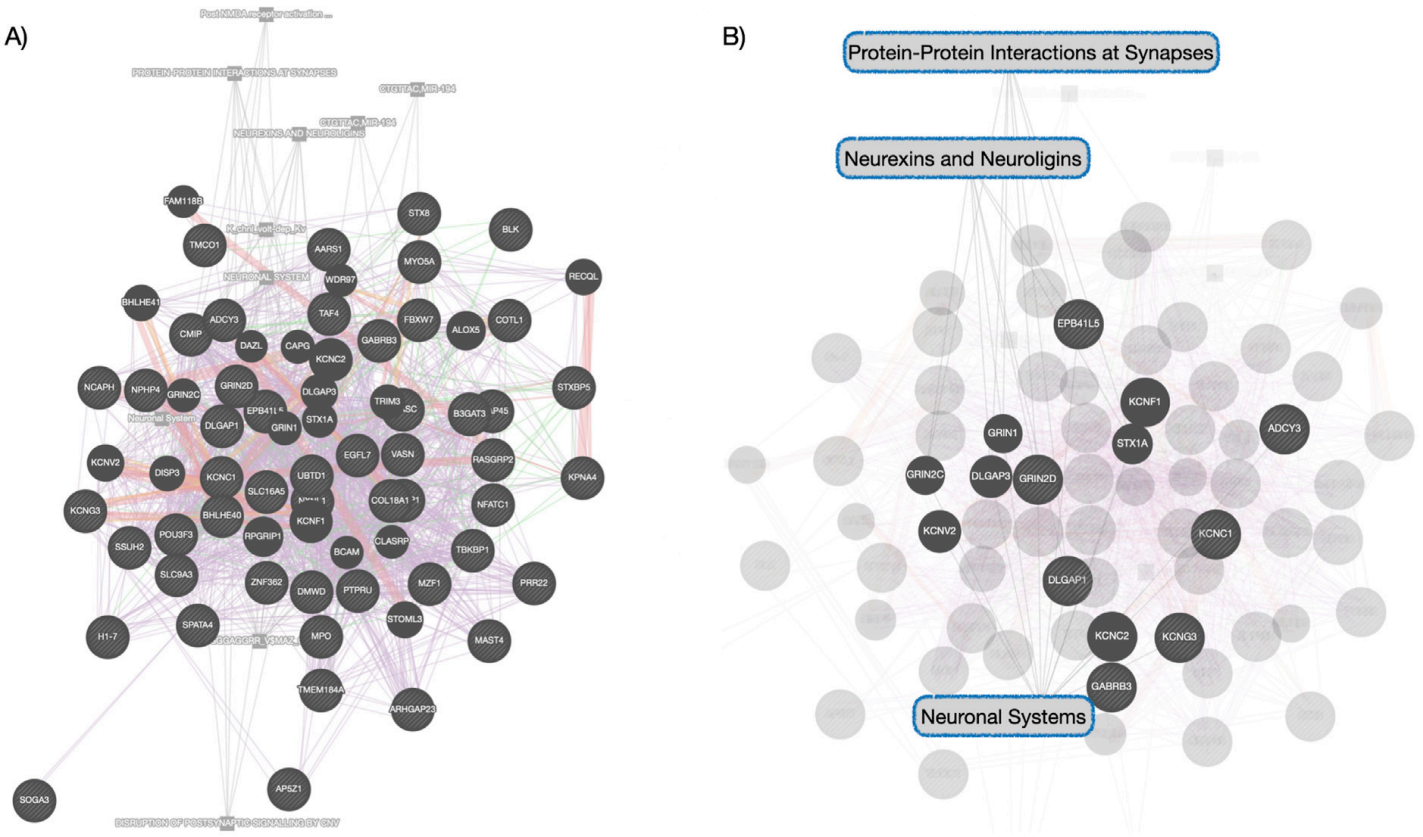
Functional Gene Network Analysis of Conserved Network—**(A)** this network shows the genes whose co-methylation patterns were conserved module across time. Different color links indicate different functional links: purple links indicate genes found in co-expression networks, red indicates protein-to-protein interactions, green indicated gene-gene interactions, orange indicates predicted protein interactions and gray indicates pathway relationships. **(B)** highlights the Reactome pathway relationships within this functional gene network analysis, consistent with the gene enrichment analysis that was performed separately.

To explore the possibility of conserved epigenetic change across time, a *post hoc* comparison between the significantly enriched Blue module in deltaT1 and significantly enriched modules in deltaT2 was done. We found 96% overlap between the Blue module in deltaT1 and the Brown module in deltaT2, with 69 of the 72 CpGs in the Blue module also included in the 270 CpGs in the Brown module. The gene enrichment analysis of these 69 CpGs revealed the conserved pathways were FBXW7 mutants, tyrosine kinase signaling in B-cells, neuronal system, voltage-gated potassium channels, neurexins and neuroligins. See Table 2 for details of the gene enrichment results of the conserved co-methylation module. Figure 2A highlights the functional gene network analysis of the conserved genes and their relationship to the gene enrichment analysis (Figure 2B) from GeneMANIA. Table 3 contains the results of the *post hoc* investigation of the CpGs involved in the neuronal-related gene enrichment pathways.

**TABLE 2:** Gene enrichment results for conserved co-methylation module.

Pathway name	Found	Ratio	FDR *p*-value
Loss of function of FBXW7 in cancer and NOTCH1 signaling	2/6	3.88^-4	0.02
FBXW7 mutants and NOTCH1 in cancer	2/6	3.88^-4	0.02
RUNX1 regulates transcription of genes in BCR signaling	2/7	4.52^-4	0.02
Neuronal system	8/490	0.03	0.02
Voltage-gated potassium channels	3/44	0.003	0.02
Neurexins and Neuoligins	3/60	0.004	0.05

**TABLE 3:** IMAGE-CpG cross-tissue correspondence results.

CpG	Gene	IMAGE-CpG average correlation
cg20227471	*ADCY3*	0.926187
cg14859324	*GABRB3*	0.8638013
cg21734356	*DLGAP1*	0.6796473
cg01483824	*GRIN2D*	0.953338
cg26703758	*KCNC1*	0.9468902
cg23167863	*EPB41L5*	0.9515299
cg22500730	*KCNG3*	0.9780846
cg14467816	*ROBO1*	0.9505681

### 3.3 Conserved co-methylation module associated with brain maturation

**FIGURE 3:**
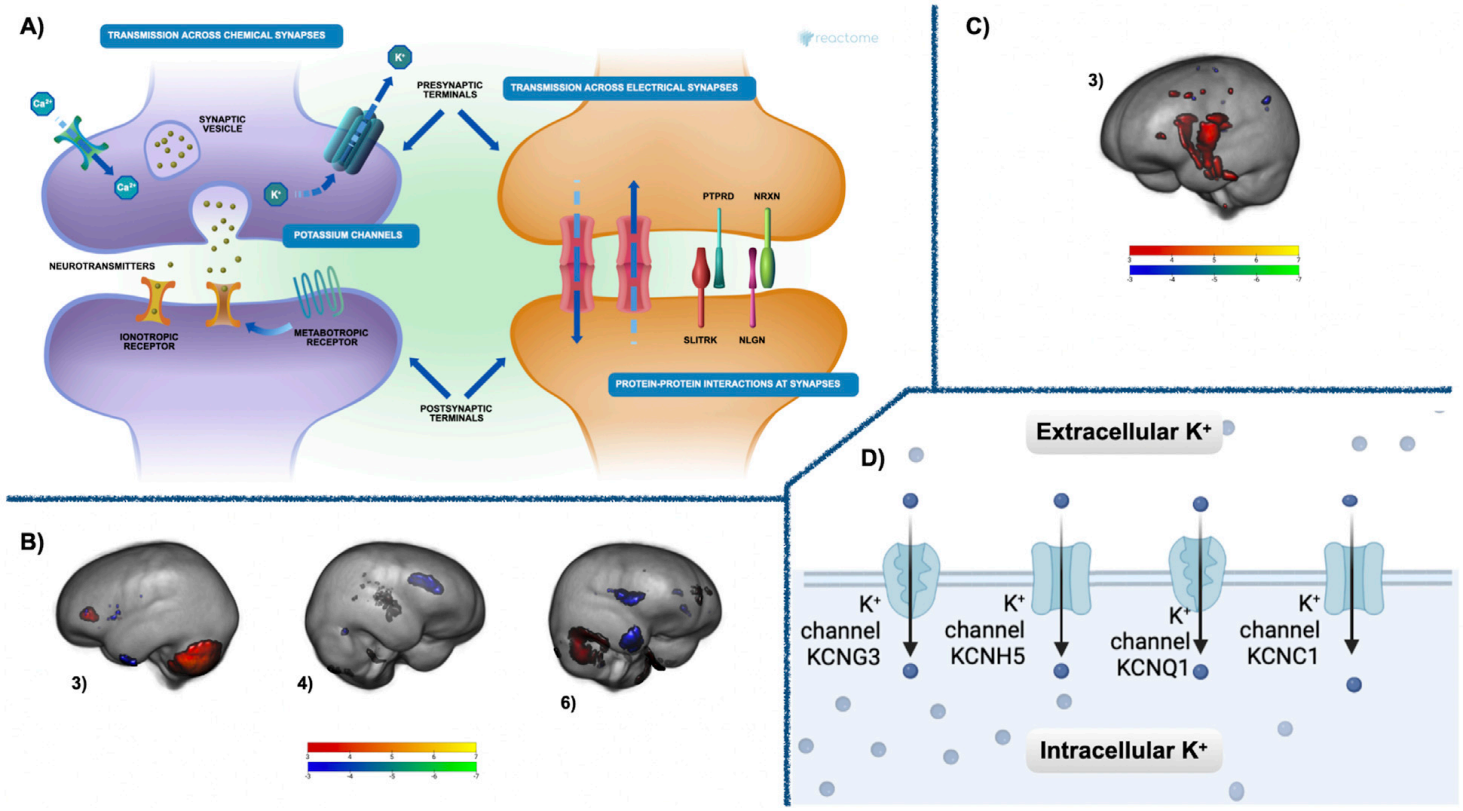
Co-methylation and brain results—**(A)** The Reactome gene enrichment results for deltaT1 Blue module and deltaT2 Brown module. The neuronal systems gene enrichment (*p* < 0.02, fdr corrected) includes pathways for chemical and electrical synapses at both pre- and postsynaptic junctions, metabolic and inotropic receptors, as well as protein-protein interactions at the synapses. See Supplementary Table 1 for a complete list of significant gene enrichment for each module. **(B)** Three components of GM maturation were significantly related in deltaT1 to the Blue module (multivariate: F = 6.55, *p* < 1.4e-6). These were components 3 (univariate: F = 9.50, *p* < 0.002), 4 (univariate: F = 26.43, *p* < 1.0e-6), and 6 (univariate: F = 19.83, *p* < 1.8e-5). **(C)** FA maturation highlighted in component 3 that was significantly related during deltaT2 to the Brown module multivariate: F = 3.92, *p* < 0.009, (univariate: F = 12.91, *p* < 8.6e-4) Both GM and FA components are thresholded from −7 < z < −3 (blue to green) and from 3 < z < 7 (red to yellow). Blue—green are areas of GM or FA decrease over time, red—yellow are areas of GM or FA increase. See Supplementary Tables 2, 3 for a comprehensive list of regions. **(D)** The Reactome gene enrichment results for deltaT1 Blue module and deltaT2 Brown module. The potassium channels included in the voltage-gated potassium channel gene enrichment pathway (*p* < 0.03, fdr corrected). See Supplementary Table 1 for a complete list of significant gene enrichment for each module.

The relationships between the co-methylation networks, GM volume, FA, and cognition were investigated using a MANCOVA analysis. Over the first change in time, deltaT1, the Blue module was significantly related (multivariate: F = 6.55, *p* < 1.4e-6) to three networks of GM volume change: Comp3 (univariate: F = 9.50, *p* < 0.002), Comp4 (univariate: F = 26.43, *p* < 1.0e-6), and Comp6 (univariate: F = 19.83, *p* < 1.8e-5). During deltaT2, the Brown module was significantly related (multivariate: F = 3.92, *p* < 0.009) to one network of FA increases, Comp3 (univariate: F = 12.91, *p* < 8.6e-4). Figure 3 highlights these networks of brain maturation and the conserved genetic pathways.

### 3.4 Unique module related to cognitive maturation

**FIGURE 4:**
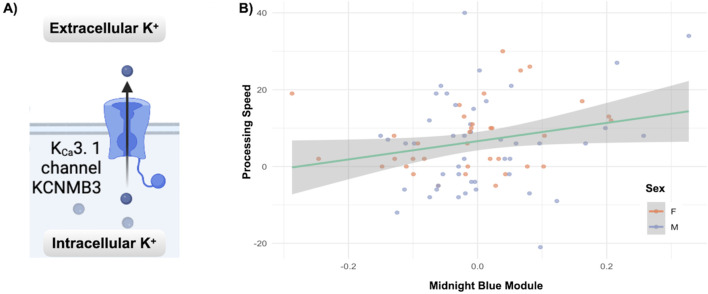
Co-methylation and cognition results: **(A)** The Reactome gene enrichment results for the Midnight Blue module from deltaT2, significantly enriched for calcium-gated potassium channels (*p* < 0.007, fdr corrected). **(B)** The relationship between increases in processing speed (PCPS) and the deltaT2 Midnight Blue module (multivariate: F = 2.66, *p* < 0.041, univariate: F = 5.04, *p* < 0.028). See Supplementary Table 1 for a complete list of significant gene enrichment for each module.

One unique module in deltaT2, the MidnightBlue module, significantly enriched for calcium-gated potassium channels, was related (multivariate: F = 2.66, *p* < 0.041) to the increase in the cognitive measure for processing speed, PCPS (univariate: F = 5.04, *p* < 0.028). Figure 4 highlights this relationship as well as the gene enrichment result.

## 4 Discussion

While the reorganization of the brain and the concurrent improvements in cognitive function that occur during adolescence have been thoroughly researched (Steinberg, 2005), little is known about the underlying epigenetic mechanisms that may be driving this phase of diverse and profound neurodevelopment. Our investigation of the co-methylation patterns across time and their relationships to brain maturation and cognitive development offers insights into possible molecular underpinnings of adolescent development. The weighted correlation analysis of the changes in DNAm showed an increase in the number of networks of co-methylation as this cohort aged. This paralleled our earlier research, in which we found dramatic decreases in methylation occurring between time points 2 and 3 for small subset of genes from this cohort undergoing changes in DNAm (Jensen et al., 2023a; Jensen et al., 2023b). To better understand the possible cause, as well as rule out that these changes might be caused by a batch effect within the DNAm data, subsamples of subjects with data from all three time points within the same batch were analyzed. The same precipitous drop in methylation between the last two time points was observed. This, coupled with an increase in the diversity of co-methylation networks in deltaT2 found in this study, suggests a biological mechanism, possibly related to pubertal status, worthy of future research. The gene enrichment analysis of the modules reflected a myriad of biological systems undergoing developmental plasticity during adolescence that include neuronal, microbiome, endocrine, immune, and cellular signaling (Gillespie et al., 2022). Despite the increase in diversity between the timepoints, 96% of the CpGs identified in the Blue module in deltaT1 were conserved within the Brown module in deltaT2. This further suggests a progression of developmental epigenetic changes, particularly since the majority of the conserved co-methylation pathways were ones associated with well-established patterns of brain and cognitive maturation.

The conserved epigenetic co-methylation patterns between the Blue and Brown modules were enriched in pathways for neuronal systems, voltage-gated potassium channels, as well as neurexins and neuroligins (Supplementary Table 1; Figure 3A). Neuronal systems within the Reactome (Gillespie et al., 2022) pathway analysis refers to gene enrichment for chemical and electrical synapses at both pre- and postsynaptic junctions, metabolic and ionotropic receptors, as well as protein-protein interactions at the synapses (Fitzpatrick et al., 2001). Potassium channels are responsible for regulating the excitability of neurons, and are expressed throughout the brain, particularly in the axon, axon nodes, axon terminals, and somatodendritic sites (McKeown et al., 2008). Neurexins and neuroligins are synaptic cell-adhesion molecules that mediate trans-synaptic signaling in excitatory glutamatergic as well as inhibitory GABAergic synapses (Craig and Kang, 2007). The functional gene network analysis also confirmed the Reactome results, showing similar significant neuronal pathways. Our *post hoc* cross-tissue analysis, using the IMAGE-CpG data set, also confirmed that the DNAm of the CpGs from saliva highlighted in these modules and significantly enriched for genes involved in neuronal process correspond strongly to the DNAm of these same CpGs in the brain. The networks of GM volume change and FA increases found in the neuroimaging analyses of this cohort are aligned with our current understanding of adolescent brain maturation (Gogtay et al., 2004; Bava et al., 2010; Tiemeier et al., 2010). The brain networks significantly related to the Blue module included GM volume increases in the cerebellum and prefrontal cortex covarying with maturation-related GM volume decreases in the frontal and occipital poles, as well as dorsal parietal cortices. One year later (deltaT2), FA increases in the middle cerebellar peduncle, the posterior limb of the internal capsule, the splenium of the corpus callosum, and the superior corona radiata, were significantly associated with the Brown module. Recent research suggests that GM volume loss measured in healthy adolescents is actually cortical thinning due to increases in axon myelination (Natu et al., 2019), possibly explaining why GM volume changes in our cohort are related to epigenetic changes in neuronal pathways in the earlier time point, followed by associations between the same epigenetic changes and FA increases later. Our previous research, focused on a small subsample of seven CpGs located on genes expressed highly in the brain, also found these same components of GM volume change and FA increases were significantly related to changes in DNAm of genes for myelination, voltage-gated potassium channels, and solute channels (Jensen et al., 2023a; Jensen et al., 2023b).

DeltaT2 also saw a significant relationship between the increase in subjects’ processing speed and the Midnight Blue module. Commonly defined as the time it takes for an individual to perceive, process, and respond to a stimulus, processing speed generally increases throughout childhood and adolescence, peaking around 15 years of age (Coyle et al., 2011). The Midnight Blue module of co-methylation patterns from deltaT2 contained enrichment for genes involved in calcium-activated potassium channels that are expressed in neurons. This distinct subfamily of potassium channel is fundamental to the regulation of neuronal excitability, being both sensitive to voltage as well as modulated by calcium (Alam et al., 2023).

One of the hallmarks of adolescent brain maturation is the change/refinement of the ratio of excitatory versus inhibitory (E/I) inputs throughout the brain (Caballero et al., 2021). This occurs through the adolescent maturation of GABAergic signaling, particularly parvabelbumin (PV)-positive interneurons, reducing the E/I ratio through an increase in inhibitory synapses (Larsen et al., 2022). Increased inhibition creates a stronger signal-to-noise ratio through suppression of spontaneous activation in local neuronal circuitry (Craig and Kang, 2007). Imbalances in either direction lead to serious neural dysfunction in the form of either hyper- or hypoexcitibilty or seizures, impairing information processing (Craig and Kang, 2007). Several of the genes and pathways experiencing changes in DNAm that were highlighted in this study may be contributing to this process. For example, the possible changes in expression of receptor subunits of *GRIN2D* and *GABRB3* due to the changes in their DNAm may be involved, but synergies between other genes could also be at play. Neurexins and neuroligins regulate GABAergic synaptogenesis, shape synaptic plasticity and efficacy in both excitatory and inhibitory synapses (Südhof, 2008; Boxer and Aoto, 2022). Changes in the DNAm of genes involved in neurexins and neuroligins, as one example, may be part of the complex orchestration of adolescent brain maturation.

While more research remains to be done to directly connect the changes in DΝΑm of the genes found in these co-methylation modules related to brain and cognitive development in adolescence, the changes occurring in the associated neural systems are well understood. Synaptic pruning, increased myelination, and the shifts in connectivity that result in a more dynamic and efficient brain (Spear, 2013) would seem to require changes in gene expression in the pathways found in our analysis. Studies in mammalian neuronal development also highlight an interconnectedness between myelination and potassium channels (Zhou et al., 1998), with clustering of the potassium channels determined by the extent of myelination present, both contributing synergistically to the excitability of the neuron (Rasband and Peles, 2016). The role calcium-activated potassium channels play in synaptic plasticity as part of a calcium modulation feedback loop (Kim and Hoffman, 2008) could explain why DNAm changes in this gene enrichment pathway were related to improvements in cognitive performance in our study.

## 5 Limitations

Stage of puberty could not be included in this study because there was no measure of hormonal change available. Future researchers should include this essential marker of adolescent development to ensure the completeness of the model. The imbalance in subjects between deltaT1 and deltaT2 was due to attrition, which is not an uncommon problem in longitudinal studies. Although our results are still informative despite this, replication with more subjects would be important going forward. Also, our understanding of what effect these changes in methylation will have on downstream gene expression is still limited (Mangiavacchi et al., 2023), but this study offers many targets for future research into the epigenetic drivers of adolescent development.

## 6 Conclusion

Understanding how changes in DNAm might be driving the changes in adolescent neural development is still a fairly unexplored field. Our research, while exploratory, indicates that there are dynamic relationships between correlated networks of methylation change and adolescent brain and cognitive development. These relationships between DNAm changes in pathways enriched for neuronal systems, potassium channels, neurexins and neuroligins and patterns of grey and white matter maturation, as well as improvements in subjects’ processing speed performance across time provide a first look at epigenetic drivers of neuronal and cognitive development in adolescence.

## Data Availability

The original contributions presented in the study are publicly available. This data can be found here: accession number GSE284550, https://www.ncbi.nlm.nih.gov/geo/query/acc.cgi?acc=GSE284550.
