# Supplementary material for: Co-methylation networks associated with cognition and structural brain development during adolescence
Source: Front Genet. 2025 Jan 7;15:1451150. doi: 10.3389/fgene.2024.1451150 (PMC11746905; doi:10.3389/fgene.2024.1451150)
Supplement: Supplementary file 1 [file DataSheet1.pdf]

Supplementary Table 1: Complete Gene Enrichment Results from Reactome

Pathway Name	Found	Ratio	FDR P-value
**Gene Enrichment for Conserved Co-methylation from DT1 to DT2**			
**Loss of Function of FBXW7 in Cancer and NOTCH1 Signaling**	2/6	3.88^-4	0.02
**FBXW7 Mutants and NOTCH1 in Cancer**	2/6	3.88^-4	0.02
**RUNX1 regulates transcription of genes involved in BCR signaling**	2/7	4.52^-4	0.02
**Neuronal System**	8/490	0.03	0.02
**Volgate-gated Potassium channels**	3/44	0.003	0.02
**Neurexins and neuroligins**	3/60	0.004	0.05
**Gene Enrichment for deltaT1 Blue Module**			
**Nuclear Receptor transcription pathway**	9/86	0.006	8.1^-8
**Voltage gated Potassium channels**	4/44	0.003	0.007
**Loss of Function of FBXW7 in Cancer and NOTCH1 Signaling**	2/6	3.88^-4	0.02
**FBXW7 Mutants and NOTCH1 in Cancer**	2/6	3.88^-4	0.02
**Neuronal System**	8/490	5.03^-4	0.02
**RUNX1 regulates transcription of genes involved in BCR signaling**	2/7	5.28^-4	0.02
**Gene Transcription pathway**	17/1586	0.104	0.03
**Potassium Channels**	4/107	0.007	0.04

Pathway Name	Found	Ratio	FDR P-value
**Gene Enrichment for deltaT2 Brown Module**			
**Signaling by FGFR2 IIa TM**	5/24	0.002	0.008
**FGFR2 mutant receptor activation**	6/43	0.003	0.008
**Voltage gated Potassium channels**	6/44	0.003	0.008
**Signaling by FGFR2 in disease**	6/58	0.004	0.02
**Phospholipase C-mediated cascade; FGFR2**	4/25	0.002	0.05
**FGFR2 ligand binding and activation**	4/26	0.002	0.05
**Gene Enrichment for deltaT2 MidnightBlue Module**			
**Ca2+ activated K+ channels**	2/10	6.5^-4	0.007
**cGMP effects**	2/18	0.001	0.01
**Nitric oxide stimulates guanylate cyclase**	2/35	0.002	0.02
**NFE2L2 regulating anti-oxidant/ detoxification enzymes**	2/39	0.003	0.02
**Gene Enrichment for deltaT2 Blue Module**			
**Nuclear Receptor transcription pathway**	8/86	0.006	0.01
**Gene Enrichment for deltaT2 Turquoise Module**			
**Nuclear Receptor transcription pathway**	12/86	0.006	0.003
**Gene Enrichment for deltaT2 Cyan Module**			

Pathway Name	Found	Ratio	FDR P-value
**Proton/oligopeptide cotransporters**	2/9	5.8^-4	0.01
**Gene Enrichment for deltaT2 Tan Module**			
**FGFR2 mutant receptor activation**	6/38	0.003	7.7^-8
**Signaling by FGFR2 in disease**	6/49	0.004	1.5^-7
**Signaling by FGFR2 IIa TM**	5/23	0.002	1.5^-7
**Phospholipase C-mediated cascade; FGFR2**	4/21	0.002	6.3^-6
**FGFR2 ligand binding and activation**	4/23	0.002	7.5^-6
**PI-3K cascade;FGFR2**	4/26	0.002	1^-5
**SHC-mediated cascade;FGFR2**	4/28	0.002	1.2^-5
**FRS-mediated FGFR2 signaling**	4/29	0.002	1.2^-5
**Downstream signaling of activated FGFR2**	4/35	0.003	2.2^-5
**Negative regulation of FGFR2 signaling**	4/37	0.003	2.5^-5
**PI3K cascade**	4/50	0.004	7.5^-5
**IRS-mediated signaling**	4/55	0.005	8.5^-5
**Signaling by FGFR2 amplification mutants**	2/2	1.7^-4	8.9^-5
**IRS-related events triggered by IGF1R**	4/59	0.005	9.6^-5
**Activated point mutants of FGFR2**	3/19	0.002	9.7^-5
**Insulin receptor signaling cascade**	4/61	0.005	9.7^-5
**IGF1R signaling cascade**	4/62	0.005	9.7^-5

Pathway Name	Found	Ratio	FDR P-value
**Signaling by Type 1 IGF1R**	4/63	0.005	1.0^-4
**Constitutive signaling by aberrant PI3K in Cancer**	4/89	0.008	3.1^-5
**Signaling by Insulin receptor**	4/89	0.008	3.1^-4
**Gene Enrichment for deltaT2 Red Module**			
**Regulation of cortical dendrite branching**	3/44	2.6^-4	7.1^-4
**Cell-cell communication**	7/165	0.01	0.01
**Cell-cell junction organization**	5/99	3.6^-4	0.03
**Gene Enrichment for deltaT2 Pink Module**			
**Interferon gamma signaling**	8/177	0.01	1.1^-4
**Interferon signaling**	8/398	0.02	0.01
**Translocation of ZAP-70 to Immunological synapse**	3/30	0.002	0.01
**Phophorylation ofCD3 and TCR zeta chains**	3/33	0.002	0.01
**PD-1 signaling**	3/33	0.002	0.01
**Generation of second messenger molecules**	3/47	0.003	0.03

Supplementary Table 2: Comprehensive list of GM ICA results - Brain regions results of the ICA highlighting covarying regions of GM change within the subjects' brains over time. These regions were identified by the MNI coordinates and the Harvard-Oxford cortical and subcortical structural atlases as well as the probabilistic cerebellar atlas

Comp1			
**Area**	**volume (cc)**	**Max Values**	**MNI (x, y, z)**
**Right Crus II**	23.5	7.2	(16, -84, -38)
**Left Crus II**	10.9	6.6	(-17, -83, -40)
**Left VIIb**	3.2	5.3	(-22, -73, -46)
**Left Crus I**	3.8	4.7	(-16, -77, -31)
**RIght Crus I**	0.5	3.9	(29, -63, -34)
**Left VI**	0.3	3.6	(-28, -60, -32)
**Vermis VI**	0.1	3.6	(-2, -73, -28)
**Vermis VI**	0.2	3.5	(2, -73, -26)
**Vermis VIIb**	0.1	3.5	(-2, -69, -31)
**Vermis VIIIa**	0.1	3.2	(0, -69, -40)
**Vermis Crus II**	0.1	3.1	(2, -76, -33)
**Vermis VIIIb**	0.1	3.0	(0, -65, -41)
**Vermis VIIIa**	0.1	2.9	(1, -73, -38)
**Lateral Occiptal Cortex, inferior division**	0.1	-3.0	(52, -70, -19)
**Brain Stem**	0.1	-3.0	(-5, -39, -27)
**RIght Crus I**	0.1	-3.0	(50, -46, -38)
**Left I-IV**	0.1	-3.1	(-1, -44, -25)
**Right Crus II**	0.4	-4.0	(46, -49, -46)
**Comp2**			
**Frontal Orbital Cortex**	11.9	5.4	(-26, 34, -18)
**Frontal Pole**	20.4	5.4	(27, 38, -17)

**Frontal Medial Cortex**	11.2	5.4	(3, 52, -19)
**Subcollosal Cortex**	0.6	3.9	(-3, 24, -13)
**Paracingulate Gyrus**	1.8	3.8	(-3, 37, -10)
**Comp3**			
**Left VIIb**	8.4	6.3	(-35, -68, -53)
**Left Crus II**	4.8	5.7	(-36, -77, -45)
**Right VIIIa**	2.9	4.1	(10, -66, -52)
**Left Crus I**	2.2	4.1	(-34, -81, -34)
**Paracingulate Gyrus**	1.7	3.6	(-2, 49, -4)
**Cingulate Gyrus, anterior division**	0.9	3.5	(2, 40, -2)
**Inferior Frontal Gyrus, pars opercularis**	0.8	-3.3	(-56, 20, -3)
**Temporal Pole**	0.5	-3.9	(-36, 5, -46)
**Frontal Pole**	0.3	-3.2	(-26, 47, 4)
**Caudate, left**	0.2	3.0	(-5, 14, -1)
**Subcallosal Cortex**	0.1	3.1	(-1, 13, -2)
**Temporal Fusiform Cortex, anterior division**	0.1	-3.2	(-35, 0, -50)
**Postcentral Gyrus**	0.1	-3.1	(-63, -6, 20)
**Right VI**	0.1	-3.0	(21, -63, -31)
**Comp4**			
**Cingulate Gyrus, anterior division**	4.5	-3.5	(2, 18, 33)
**Thalamus, left**	2	3.7	(-18, -25, 15)

**Cingulate Gyrus, posterior division**	1.4	3.3	(-15, -40, 35)
**Thalamus, right**	1.1	3.2	(16, -19, 15)
**Right IX**	0.4	3.9	(5, -42, -37)
**Insular Cortex**	0.4	3.4	(-29, -29, 19)
**Lingual Gyrus**	0.4	-3.1	(14, -65, -15)
**Supramarginal Gyrus, posterior division**	0.3	3.1	(42, -41, 12)
**Left IX**	0.2	3.2	(-6, -45, -38)
**Superior Parietal Lobule**	0.2	3.2	(29, -40, 41)
**Caudate, right**	0.2	3.1	(21, -28, 21)
**Paracingulate Gyrus**	0.2	-3.0	(5, 28, 38)
**Right VI**	0.2	-3.1	(17, -67, -17)
**Precentral Gyrus**	0.1	3.1	(-19, -33, 39)
**Left I-IV**	0.1	3.0	(-9, -38, -29)
**Putamen, left**	0.1	2.9	(-28, -14, 9)
**Parietal Operculum Cortex**	0.1	2.9	(45, -25, 28)
**Frontal Pole**	0.1	2.9	(31, 66, 0)
**Comp5**			
**Temporal Occipital Fusiform Cortex**	1.7	3.6	(39, -52, -12)
**Temporal Fusiform Cortex, posterior division**	1.0	-3.7	(-42, -30, -18)
**Frontal Orbital Cortex**	0.8	-3.4	(-16, 25, -18)

**Inferior Temporal Gyrus, posterior division**	0.6	3.5	(-60, -44, -22)
**Middle Temporal Gyrus, posterior division**	0.6	3.2	(-61, -29, -14)
**Temporal Pole**	0.6	-3.4	(59, 8, -24)
**Inferior Temporal Gyrus, temporooccipital part**	0.5	3.5	(-55, -56, -24)
**Planum Temporal**	0.4	3.3	(-58, -21, 8)
**Supramarginal Gyrus, anterior division**	0.3	3.2	(-57, -25, 22)
**Middle Frontal Gyrus**	0.3	-3.0	(-43, 5, 58)
**Middle Temporal Gyrus, temporooccipital part**	0.2	3.3	(41, -56, 13)
**Precentral Gyrus**	0.2	3.2	(-55, -3, 13)
**Lingual Gyrus**	0.2	3.1	(20, -56, -6)
**Right VIIIa**	0.2	3.1	(31, -37, -44)
**Hippocampus, left**	0.2	-3.2	(-37, -27, -11)
**Temporal Occipital Fusiform Cortex**	0.2	-3.2	(-30, -54, -16)
**Superior Parietal Lobule**	0.2	-3.1	(-24, -43, 69)
**Putamen, right**	0.1	3.2	(14, 4, -16)

**Temporal Fusiform Cortex, posterior division**	0.1	3.2	(35, -7, -32)
**Middle Frontal Gyrus**	0.1	3.2	(-25, -4, 48)
**Frontal Orbital Cortex**	0.1	3.0	(22, 19, -18)
**Lateral Occipital Cortex, superior division**	0.1	3.0	(23, -64, 64)
**Frontal Pole**	0.1	-3.0	(42, 37, 8)
**Lateral Occipital Cortex, superior division**	0.1	-3.1	(27, -78, 16)
**Cingulate Gyrus, anterior division**	0.1	-3.0	(9, -9, 46)
**Comp6**			
**Frontal Operculum Cortex**	3.8	-3.9	(41, 12, 7)
**Temporal Pole**	3.3	-4.0	(42, 7, -40)
**Right Crus II**	1.7	4.8	(47, -44, -44)
**Right Crus I**	1.6	4.5	(53, -47, -34)
**Inferior Temporal Gyrus, posterior division**	1.3	4.5	(56, -16, -36)
**Left VI**	0.8	3.7	(-41, -36, -35)
**Parahippocampal Gyrus, anterior division**	0.8	-3.7	(25, 5, -35)
**Left VIIb**	0.6	3.7	(-40, -43, -52)
**Frontal Pole**	0.4	3.2	(-8, 70, 14)
**Insular Cortex**	0.4	-3.3	(-33, 20, 7)

**Middle Frontal Gyrus**	0.4	-3.7	(-39, 14, 28)
**Right Crus VI**	0.3	3.4	(28, -57, -36)
**Inferior Temporal Gyrus, anterior division**	0.3	-3.5	(41, 0, -42)
**Occipital Pole**	0.2	3.0	(34, -96, -11)
**Vermis VIIIb**	0.2	-3.3	(-6, -63, -41)
**Middle Frontal Gyrus**	0.1	3.2	(26, 16, 40)
**Precentral Gyrus**	0.1	3.0	(23, -24, 56)
**Central Operculum Cortex**	0.1	-3.4	(44, 9, 7)
**Vermis IX**	0.1	-2.9	(1, -60, -44)
**Inferior Temporal Gyrus, temporooccipital part**	0.1	-3.1	(46, -43, -19)
**Frontal Pole**	0.1	-3.1	(-38, 49, 0)
**Parietal Operculum Cortex**	0.1	-3.0	(44, -32, 23)
**Left IX**	0.1	-3.0	(-5, -63, -45)
**Superior Frontal Gyrus**	0.1	-3.0	(-18, 16, 65)
**Comp7**			
**Frontal Pole**	2.5	-3.6	(-26, 58, 26)
**Subcallosal Cortex**	1.8	4.1	(-3, 13, -18)
**Paracingulate Gyrus**	1.8	-3.3	(2, 45, 24)
**Inferior Frontal Gyrus, pars opercularis**	1.6	-3.5	(55, 15, 33)

**Frontal Orbital Cortex**	1.5	3.7	(20, 13, -18)
**Inferior Frontal Gyrus, pars opercularis**	1.3	3.9	(39, 9, 22)
**Temporal Pole**	1.3	3.6	(-39, 23, -35)
**Right IX**	1.1	3.9	(8, -59, -53)
**Superior Frontal Gyrus**	1.0	-3.5	(20, 26, 60)
**Cingulate Gyrus, anterior division**	1	-3.3	(-4, 40, 18)
**Left IX**	0.9	4.1	(-5, -58, -54)
**Insular Cortex**	0.4	3.5	(31, 7, 14)
**Frontal Opercular Cortex**	0.3	3.7	(33, 10, 16)
**Inferior Temporal Gyrus, anterior division**	0.2	3.6	(-52, 1, -36)
**Paracingulate Gyrus**	0.2	3.0	(15, 16, 42)
**Temporal Fusiform Cortex, anterior division**	0.1	3.0	(26, 2, -48)
**Parahippocampal Gyrus, anterior division**	0.1	3.4	(-14, 2, -23)
**Caudate, right**	0.1	3.4	(12, -2, 26)
**Putamen, right**	0.1	3.1	(27, 1, 12)
**Subcallosal Cortex**	0.1	3.0	(0, 12, -11)

Supplementary Table 3: Comprehensive list of FA ICA results - Brain regions results of the ICA highlighting covarying regions of FA change within the subjects' brains over time. These regions were identified by the MNI coordinates and the Harvard-Oxford cortical and subcortical structural atlases as well as the probabilistic cerebellar atlas.

**Component 1**			
**Area**	**volume (cc)**	**Max Value**	**MNI (x, y, z)**
**Middle Cerebellar Peduncle**	0.8	7.4	(22, -38, -36)
**Precentral Gyrus**	0.7	5.2	(21, -20, 63)
**Angular Gyrus**	0.1	4.9	(-38, -58, 35)
**Precuneus Gyrus**	0.4	4.7	(-18, -62, 36)
**Temporal Fusiform Gyrus, anterior division**	0.3	4.1	(-39, -4, -26)
**Angular Gyrus**	0.1	4.0	(-42, -56, 36)
**Genu of Corpus Callosum**	0.3	3.9	(-3, 30, 5)
**Lateral Occipital Cortex, superior division**	0.2	3.9	(-39, -60, 39)
**Frontal Orbital Cortex**	0.1	3.7	(39, 34, -2)
**Precuneus Gyrus**	0.1	-3.7	(13, -55, 40)
**Superior Parietal Lobule**	0.1	-3.7	(27, -56, 45)
**Temporal Fusiform Gyrus, anterior division**	0.1	-3.9	(36, -3, -35)
**Posterior corona radiata L**	0.6	-4.2	(-26, -54, 21)
**Postcentral Gyrus**	0.1	-4.5	(-19, -33, 58)
**Precentral Gyrus**	0.3	-4.5	(-28, -25, 51)

**Inferior Temporal Gyrus, anterior division**	0.1	-4.7	(39, 3, -34)
**Lateral Occipital Cortex, superior division**	0.2	-4.8	(-19, -60, 52)
**Frontal Orbital Cortex**	0.2	-5	(14, 18, -23)
**Cerebellum, Left VIIIa**	0.3	-5.1	(-24, -61, -46)
**Subcallosal Cortes**	0.1	-5.2	(-8, 13, -23)
**Frontal Pole**	1.1	-5.3	(-18, 44, -12)
**Inferior Temporal Gyrus, posterior division**	0.1	-6.1	(-48, -19, -28)
**Cerebellum, Right VIIb**	0.8	-6.8	(30, -62, -46)
**Component 2**			
**Posterior Corona Radiata L**	0.9	4.6	(-29, -30, 28)
**Anterior Corona Radiata R**	0.5	5.2	(17, 22, 26)
**Body of Corpus Callosum**	4.1	4.9	(-15, 5, 33)
**Posterior Thalamic Radiation L**	0.1	3.5	(-28, -60, 11)
**Splenium of Corpus Callosum**	0.1	4.8	(17, -41, 10)
**Genu of Corpus Callosum**	0.3	4.8	(-14, 20, 26)
**Middle Frontal Gyrus**	0.2	3.6	(-35, 20, 36)

**Superior Longitudinal Fascisulus L**	1.0	-5	(-33, -34, 32)
**Superior Corona Radiata R**	0.4	-4.2	(24, 6, 30)
**Superior Fontal Gyrus**	0.1	-4.1	(23, 8, 55)
**Postcentral Gyrus**	0.1	-4	(-30, -37, 50)
**Temporal Fusiform Cortex, posterior division**	0.1	-3.8	(-37, -27, -19)
**Precentral Gyrus**	0.1	-3.8	(8, -16, 63)
**Superior Parietal Lobule**	0.1	-3.8	(-24, -52, 49)
**Component 3**			
**Middle Cerebellar Peduncle**	0.2	4.2	(19, -38, -33)
**Posterior Limb of Internal Capsule L**	0.9	4.0	(-19, -13, 0)
**Splenium of Corpus Callosum**	0.5	4.1	(2, -37, 12)
**Posterior Limb of Internal Capsule R**	0.1	3.5	(17, -8, -1)
**Superior Corona Radiata L**	0.1	3.9	(-28, -18, 19)
**Lateral Occipital Cortex, superior division**	0.1	-3.6	(-36, -62, 34)
**Component 4**			

**Splenium of Corpus Callosum**	2.5	5.6	(-24, -56, 11)
**Tapetum R**	0.7	4.5	(27, -49, 20)
**Cuneal Cortex**	0.3	4.9	(17, -83, 26)
**Lateral Occipital Cortex, superior division**	0.3	4.0	(-36, -60, 33)
**Body of Corpus Callosum**	0.1	3.6	(-10, -14, 28)
**Posterior Thalamic Radiation**	0.1	3.5	(-29, -76, 4)
**Precentral Gyrus**	0.1	-3.9	(13, -18, 68)
