# Supplementary figures and images for: Co-methylation networks associated with cognition and structural brain development during adolescence
Source: Front Genet. 2025 Jan 7;15:1451150. doi: 10.3389/fgene.2024.1451150 (PMC11746905; doi:10.3389/fgene.2024.1451150)

**A)**

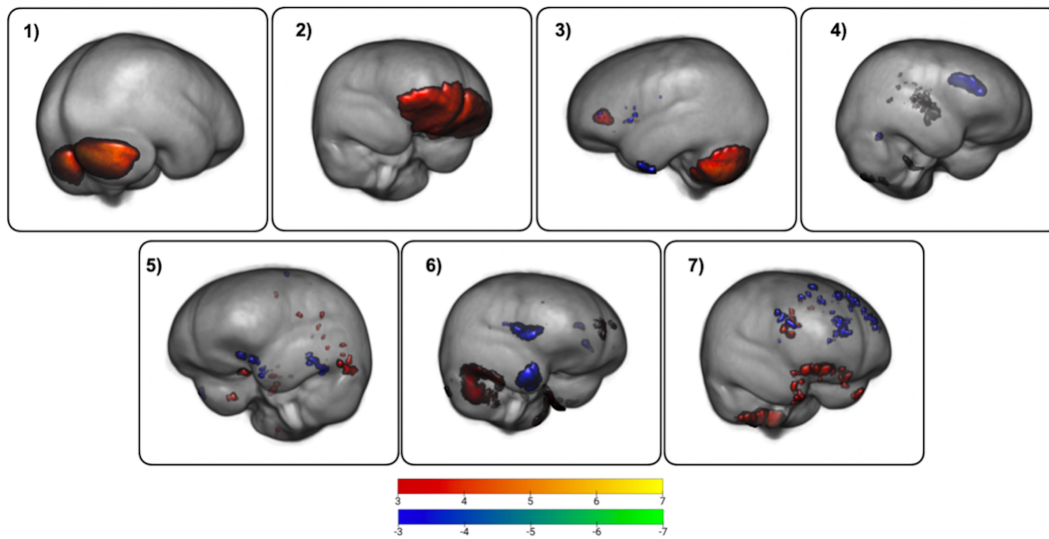

**B)**

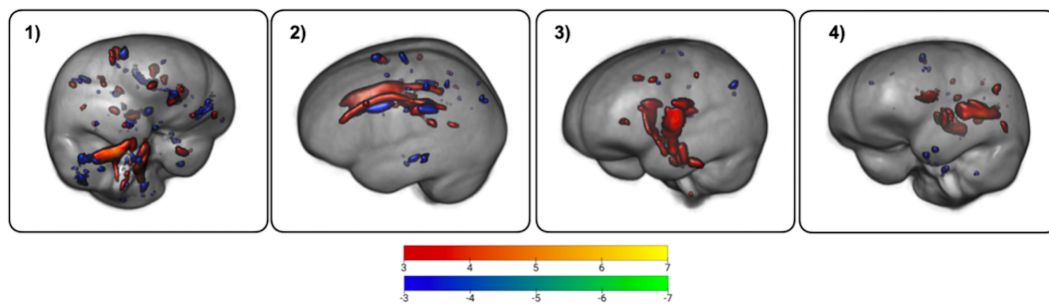

**C)**

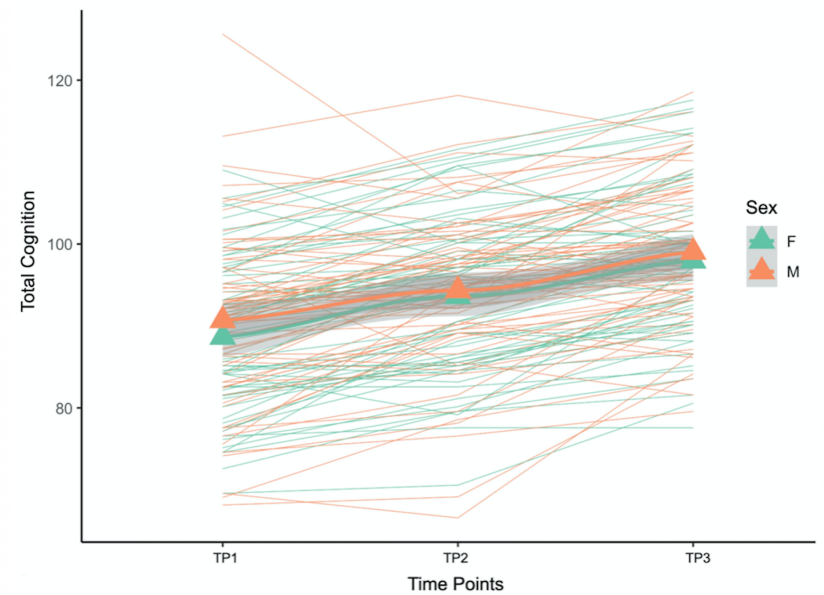

Supplement: Supplementary file 2 [file Image1.pdf]
